# The FHJ debate: There is no evidence for the drive towards virtual wards

**DOI:** 10.1016/j.fhj.2026.100544

**Published:** 2026-06-25

**Authors:** Tim Cooksley, Kyaw Myat Thu

**Affiliations:** a Acute Medicine, The Christie and Manchester University NHS Foundation Trust, United Kingdom; b Medicine for Older People and Stroke Medicine, Frimley Health Foundation Trust, United Kingdom

**Keywords:** Virtual wards, Hospital at Home, Evidence-based medicine, Patient experience, Scientific evidence

## Abstract

Virtual wards and hospital at home are rapidly growing models of care globally and in the UK. Evidence is growing around positive impact of specialist-led hospital at home care for frail, multimorbid patients in comparison to traditional hospital admission for treatment of certain medical conditions. However, the scope of practices under the term ‘virtual wards’ varies, resulting in unclear quantitative results. This themed article discusses differing viewpoints – are virtual wards inconsistent distractions during NHS crisis, or patient-centred innovations for future healthcare?

## In favour of the motion

*Tim Cooksley*

NHS urgent and emergency care (UEC) is under unbearable strain; strain that is increasingly causing harm to patients.^1^, ^2^ Emergency department (ED) overcrowding and stasis of flow through acute medical units with temporary escalation spaces prevent timely and high-quality care.^3^, ^4^ This has led to low public confidence, and the media remain full of stories featuring images of patients in corridors, long queues of ambulances outside EDs and horrific patient stories.^5^ Low staff morale, perpetuating burnout, high sickness rates and reducing productivity accentuate the problem.

At the heart of the crisis remains the fundamental problem that demand has exceeded capacity in all elements of healthcare delivery for a significant period. One of the 10 high-impact interventions described by NHS England and a key element of the recovery plan is an increase in the number of virtual ward beds.^6^

Virtual wards are not new. They have been used in various guises and models, both in the UK and internationally, for decades. The heterogeneity of their description and implementation is broad and thus their evaluation is immensely challenging. This had been complicated further by encompassing hospital at home services in the latest definition of virtual ward. Thus, a ‘virtual ward’ could vary from telephone monitoring using no technological innovations to a highly innovative sophisticated hospital at home delivering complex therapies in the patient’s home, supported by point-of-care ultrasound and telemedicine.^7^, ^8^, ^9^

A ‘virtual ward bed’ is used to supposed to be used for a patient who can be managed in their own home, whose care would traditionally have been delivered in an inpatient setting. Thus, essential components of hospital care, including senior clinician decision making, multidisciplinary assessment and rehabilitation, physiological monitoring and delivery of intravenous treatments, must form part of the service.^10^ Workforce requirements to deliver this level of care at home are significant, and shortages continue to be a barrier to the delivery and expansion of virtual wards that utilise a hospital at home model and meet the defined criteria.

There has been a failure to evaluate and monitor the proportion of patients treated on a virtual ward bed who had inpatient hospital requirements and would have otherwise needed a bed. There have been large numbers of patients added to a ‘virtual ward bed’ who would traditionally have already been managed at home, thus utilising resources less than optimally. Increased monitoring and clinical reviews to develop virtual wards, rather than identifying clinical cohorts who could benefit, has become a huge issue. If patients are well enough to be at home without any acute intervention, the clinical rationale for remote monitoring is very limited.^10^

This challenge was noted in the National Institute for Health and Care Research (NIHR)-funded analysis of the 10 high-impact initiatives, concluding that the principal limitations affecting the evidence base were due to the categorisation of interventions and headline outcome.^6^ Further, and crucially in this debate, they noted that only the hospital at home element of the virtual ward programme had a sufficiently robust definition to support evaluation.^6^ The myriad of other ‘virtual wards’ have no evidence to suggest that they add any value, and the continued decline in UEC performance supports the opinion that they are not a useful component of recovery.

The shortages in social care, with growing demand, short staffing and continued underinvestment, remain at the heart of the UEC crisis in England. The number of patients in hospitals with no criteria to reside continues to sit at around 15% of acute and general beds. The delays for patients waiting for long-term care (as illustrated by the rising number of 21+ days length of stay) continues to rise. Virtual wards offer no support or solutions for this cohort of patients. A Norwegian study supported this finding, showing benefit of community virtual wards for mildly frail patients, but skilled nursing facilities were essential for frailer individuals.^11^ Most patients stranded in hospital in England are frail.

There is nothing virtual about patients lying in corridors and temporary escalation spaces. Many are older, and delay-related harm is occurring on an hourly basis. The demand for acute care will continue to rise and pressures are at unsustainable levels. Hospital at home services add an innovative, resource-intense solution for some patients. The myriad of other virtual ward models do not. They are an increasing distraction from the need for urgent long-term workforce, clinical and capacity plans that are essential to rebuild UEC and the wider NHS alongside the imperative need for investment in, and development of, a social care strategy.

## Against the motion

*Kyaw Myat Thu*

Virtual wards (VW) and hospital at home (HaH) originate from one core innovative concept – ‘we can adapt to provide safe hospital-level care in the community’. This is an audacious idea to risk-averse medical minds, attracting frequent criticism about lacing evidence. So, what is evidence? Evidence-based medicine (EBM), first formally developed in 1967, envisioned to apply scientific data to improve diagnosis and treatment of patients. EBM stands on three main dimensions: clinical experience, patient value and best research evidence.^12^ While EBM enables medicine to be standardised and science-based, it drives clinicians to focus more on data evidence than patient-centred care or clinical experience perspectives of EBM.

HaH and VW are newer branches of hospital medicine, providing acute medical care beyond traditional brick-and-mortar hospital walls. While VWs answer to many names (‘hospital at home, home hospital, virtual wards or acute care at home’), they serve one simple purpose: to provide hospital-level acute treatment, investigation and monitoring in the community. Hospital at home (HaH) was voted as the internationally accepted name for ‘acute care in community’ at the World Hospital at Home Congress 2023, and the British Geriatrics Society supports the name ‘hospital at home’ over ‘virtual wards’.^13^ NHS England uses both names interchangeably. I will use the name virtual wards (VW) in this article for the sake of argument title.

While HaH has been established in Scotland since 2010, VWs came into clinical practice in England during the COVID pandemic, initially as an alternative measure to provide hospital care to our most vulnerable patients. Momentum and success of VWs had accelerated beyond COVID-related care over the last 5 years, with many NHS trusts developing a variety of models and capacities – from home-based monitoring with telemedicine to bedside point-of-care investigations, clinical diagnosis and treatment. A dedicated UK HaH society is now available to support diverse practice of different VWs in the nation. NHS hospital trusts developed a variety of VW models operated by different specialist teams. Although operational settings, approaches and resources vary among different NHS trusts as well as globally, the core of VWs is to provide hospital-level care, monitoring and treatment in the community.

Approaches of VWs vary among different NHS hospitals, resulting in limited large-scale unifying data to draw scientific conclusions to provide simple numerative evidence, especially for the virtual monitoring model. However, VWs delivering hospital-level care possess an abundance of patient experience-based research and evidence.^14^ A recent review of nine randomised controlled trials reveals that acute treatment at home is as safe as traditional hospital care for selected patients, with higher patient and satisfaction for different health conditions.^15^, ^16^ Patient-centred care is one of three main pillars on which evidence-based medicine is built and thus, research has shown that VWs follow EBM concepts.

With increasing awareness of hospital-associated harm in frail older patients, newer quantitative studies from single-centre home treatment VWs are comparing not only safety but also primary and secondary outcomes, including hospital-associated harm such as delirium.^17^ These studies are showing significant reduction in hospital-associated harm in older frail people who received acute care in the community against traditional hospital admission for conditions such as pneumonia or urinary tract infections. This is potentially practice-changing evidence for emergency and acute medical care of frail patients in the future at the hospital front door, or even at pre-hospital triage stage.

Acute hospital-level care at home with VWs is an international health care movement expanding rapidly on a global scale.^18^ The World Hospital at Home Congress is a multinational conference for multidisciplinary practitioners working with VWs sharing guidance, network support and scientific lectures, including the latest research. The establishment and content of this large international conference demonstrates that VWs are accepted worldwide and incorporated into clinical practice.

It is obvious that not every acutely unwell patient is suitable to be treated in the community. However, for appropriate patients, VWs can provide tailored treatment in the community and prevent negative healthcare-associated harms. Although VWs are not a magic wand for reducing NHS pressures, current evidence has shown that VWs are a golden armour of person-centred care for vulnerable and complex patients who need acute hospitallevel care.

While VWs have yet to provide simple open-pack evidence with excellent numbers needed to treat (NNT), like mechanical thrombectomy for stroke, many research outcomes have shown positive patient and care-provider experience complying with EBM. Moreover, increasing numbers of quantitative studies in HaH model VWs are signalling a trajectory of more robust scientific evidence soon. So far, data have shown that VWs with acute treatment at home improve care, patient experience of healthcare and support clinicians by enabling treatment of the right patients in the right place at the right time.^19^ In my view, we are arguing a redundant question today; instead, we should be asking ‘How do we provide the best standard VWs services to our patients?’.

## CRediT authorship contribution statement

**Cooksley Tim:** Writing – review & editing, Writing – original draft, Resources, Methodology, Conceptualization. **Thu Kyaw Myat:** Writing – original draft, Resources, Methodology, Conceptualization.

## Funding

This article did not receive any specific grant from funding agencies in the public, commercial, or not-for-profit sectors.

## Declaration of competing interest

The authors declare that they have no known competing financial interests or personal relationships that could have appeared to influence the work reported in this paper.
